# Five members of a mixed-sex group of bottlenose dolphins share a stereotyped whistle contour in addition to maintaining their individually distinctive signature whistles

**DOI:** 10.1371/journal.pone.0233658

**Published:** 2020-05-22

**Authors:** Brittany L. Jones, Risa Daniels, Samantha Tufano, Sam Ridgway

**Affiliations:** National Marine Mammal Foundation, San Diego, CA, United States of America; Institute of Deep-Sea Science and Engineering, Chinese Academy of Sciences, CHINA

## Abstract

Most commonly, animal communication systems are driven by shared call repertoires, with some individual distinctiveness encoded as a byproduct of voice cues. We provide evidence that bottlenose dolphins produce both individually distinctive whistles, and a shared whistle type. A stereotyped whistle contour (termed the group whistle) is shared by five bottlenose dolphins that have lived, worked, and traveled together for at least 21 years. These five dolphins are members of a group of eight dolphins that work as a specialized team for the Navy Marine Mammal Program. Each dolphin is routinely recorded during periods when an individual is isolated from the others in above ground pools as part of their routine training. Each of the eight dolphins has an individually distinctive signature whistle. In addition, at least five of these dolphins share a distinct non-signature whistle type. This shared whistle contour was produced an average of 22.4% +/- 9.0% of the time during periods in which individuals were isolated. During these isolations the signature whistle was produced an average of 42.9% +/- 11.9% of the time. This is consistent with decades of signature whistle research. A group of 10 naïve observers rated the similarity of the different whistle contours. The observers rated the group whistle contour produced by all five dolphins as highly similar (P < 0.01). Their ratings further showed that the signature whistles of the five dolphins were very different (P < 0.01). These findings were further supported by discriminant function analyses. That said, the shared whistle contours still exhibited individual differences which may allow conspecifics to identify the producer even when a whistle contour is shared among multiple dolphins. This is the first in-depth analysis of a non-signature whistle type shared among multiple conspecifics.

## Introduction

Shared call types have been recorded in a diverse group of taxa such as birds [1–6], bats [7], primates [8,9], and marine mammals [10–14]. Song birds use a system of song sharing to manage social relationships and maintain territories [15,16]. For example, sparrows share songs with their neighbours and will use shared song types when responding in an interaction [17,18]. When song birds are being territorial they can escalate an interaction by matching the shared song type that was emitted by their adversary [19]. In non-aggressive interactions, the respondent may choose to produce a shared song type that differs from the original callers’ [17,18]. The other common occurrences of shared repertoires are found in dialects. Dialects are vocal repertoires of groups of animals (e.g., killer whales, sperm whales) developed through vocal production learning. The proportion of shared call types, and the similarity of the acoustic characteristics of specific call types are higher between animals that live and interact with one another [20]. This creates an overlap in the repertoires of a population or species, with smaller groups or matrilines’ having more similar call repertoires within groups than between [21].

To date there have been a number of potential explanations for why such a diverse array of species use shared vocal repertoires. The password hypothesis suggests that calls serve as badges or passwords which allows the producer access to the group benefits (e.g., resources, territory, mating opportunities) [7,22–25]. The group cohesion hypothesis suggests that the shared calls purpose is to maintain group cohesion and coordinate group behaviours as opposed to excluding non-group members [25,26]. The affiliative hypothesis proposes that the process of forming group specific calls is a signal of the animal’s investment in the relationship [26,27]. Dahlin et al., [25] founded the social association hypothesis which suggests that the selective use of shared call types can help manage changing social relationships, which is similar to the communication accommodation theory in humans [28–30]. Shared call characteristics also can increase communication efficiency by gaining the attention of the call receiver, especially in noisy environments [23,24,26,27,31–33].

While both individually distinctive vocal types, and shared calls may provide benefits to social species, it is likely that having the ability to use both to some degree would be most beneficial. Kremers et al., [34] suggests that being individually recognizable is likely socially important even for species that share group calls.

For over 50 years researchers have recorded signature whistles produced by bottlenose dolphins in both the wild and captivity. Signature whistles are the whistle type recorded most commonly from a dolphin when isolated [35]. This whistle type is unique for each dolphin and broadcasts the individual’s identity to conspecifics [14]. A signature whistle copy can be produced by a non-owner which some have suggested may allow the producer to address or elicit the attention of the whistle owner [27]. Signature whistles typically comprise between 47–100% [12] of the whistles produced when a dolphin is isolated, and between 38–70% of all whistles produced by free swimming animals [36]. Copies of conspecifics’ signature whistles are quite rare, only occurring between 0.007–0.180 copies a minute in dolphins recorded in captivity or during capture-release events [14,37]. That said, copying rates in free-swimming dolphins is less clear as Quick and Janik [38] did not record any copying events during meetings of free-ranging groups of dolphins but King et al., [39] recorded whistle matching (including non-signature whistle matching) during cooperative mate guarding events in the wild. Although a relatively large percentage of a dolphin’s vocal repertoire consists of non-signature whistle types (commonly referred to as variant whistles), very little is known about these vocalizations [40,41]. For example, Sayigh et al., [41] recorded non-signature whistle responses during experimental playbacks, and found a general whistle contour shape (i.e., M-shape) that was produced by four different subjects.

A few studies have reported that dolphins share whistle types with conspecifics. In 1986, Tyack [42] reported that two male dolphins who were housed together each produced their own signature whistle, and the other dolphin’s signature whistle. He found that the non-owner produced the other dolphin’s signature whistle about 20% of the time which is much higher than other reports of signature whistle copying [37]. Watwood et al., [12] suggested that the two males in Tyack’s (1986) study [42] may have simply developed a shared whistle repertoire which included mostly these two whistle types. Watwood et al., [12] identified shared whistle types in wild bottlenose dolphins in Sarasota Bay, Florida. The authors found that up to 25% of the dolphins’ whistle types were shared with other members of the population. This percentage was even higher in allied male dolphins. Janik and Slater [14] found that when a group of dolphins were swimming all together, they primarily produced non-signature whistles, and that these were commonly produced by all groupmates. Finally, two studies found evidence of bottlenose dolphins producing shared non-signature whistles during cooperative behaviors (i.e., foraging [43] and mate guarding [39]).

We present evidence that a group of bottlenose dolphins rely heavily on their individualized signature whistles, but also commonly produce a shared whistle type during periods of separation. Further we explore whether shared whistle contours also encode individually distinct information.

## Materials and methods

The U.S. Navy Marine Mammal Program (MMP) houses and cares for a population of dolphins in San Diego Bay, CA (32.6717° N, 117.1441° W). The MMP is AAALAC-accredited and adheres to the national standards of the United States Public Health Service Policy on the Humane Care and Use of Laboratory Animals and the Animal Welfare Act. The MMP’s animal care and use program is routinely reviewed by an institutional animal care and use committee (IACUC) and the Navy Bureau of Medicine and Surgery (BUMED). BUMED concurred with the approval of MMP IACUC protocol #130–2018 and assigned NRD#1134 to the protocol for this project.

The focal group is made up of eight bottlenose dolphins (4 females and 4 males). These eight dolphins are considered a designated group, as they make up one of the specially trained teams that live, work, and travel together as part of the MMP. Members of this group of animals spend significantly more time together and in acoustic contact with one another than they do with the other members of the larger population. Group membership is defined as having been assigned to this working team in which the team members live, work, and travel together. This particular team of eight dolphins has been working together for at least seven years, with some members having worked on the same team for up to 28 years (Table 1). The five dolphins that are included in this study (i.e., they have been recorded producing the group whistle a minimum of 15 times) have all been a part of this delineated group for 21 years.

**Table 1:** Characteristics of the 5 focal dolphins.

Dolphin	Sex	Age (years)	Acquisition Wild Caught Or Captive Born	Region of Acquisition or First Year of Life	Group Membership (years)	Shared Whistle Contour Recorded?
CHE	F	41	WC	Gulf of Mexico (MS)	28	Yes
CST	F	18	CB	San Diego Bay (CA)	7	Yes
						*3 times
EVN	M	22	CB	San Diego Bay (CA)	10	No
KOA	M	28	CB	Oahu (HI)	21	Yes
LOK	M	22	CB	San Diego Bay (CA)	10	No
PUN	F	38	WC	Gulf of Mexico (MS)	24	Yes
TEN	F	36	WC	Gulf of Mexico (MS)	28	Yes
SPE	M	39	WC	Gulf of Mexico (MS)	24	Yes

On any given day, in their off-time, the focal dolphins are housed in natural seawater pens with up to 14–20 other same-sex dolphins. Although the males and females reside separately in same-sex groups during non-work hours, they are separated only by 30 feet of seawater, and are therefore almost always in acoustic contact. These pods can change to mimic the fission-fusion society of bottlenose dolphins per management’s discretion.

Six out of the eight members of this group have been recorded producing the group whistle contour, but one dolphin did not produce it frequently enough to meet the minimum requirement to be included in subsequent analyses (N = 15). Two other team-members have not been recorded producing the group whistle contour to date. Therefore, we include data from five focal dolphins (3 females, 2 males) (Table 1).

Although this group is artificially formed by management, we have over 204 hours of recordings of 44 other dolphins (M = 4.6 hours/dolphin) at the MMP. A total of 5948 whistles have been recorded from these non-focal dolphins during periods in which individuals were isolated from all of its groupmates. To date, we have not recorded any other dolphin producing this shared whistle contour. While it is absolutely possible that other dolphins at the MMP have and/or still do produce the shared whistle contour, it is unlikely that it is a population-wide shared contour and seems to be most commonly used by members of this particular group.

Individual dolphins were recorded during periods of isolation from group mates while free swimming in an above ground pool as part of their routine training procedures between August 2018 and August 2019. Solo recordings ensured that the focal dolphin produced all of the recorded whistles. The goal of the recordings was to identify the signature whistles and vocal repertoire of this group of dolphins as part of a larger project.

All of the whistles were recorded with one of the following systems: a self-calibrating single-channel Soundtrap (Ocean Instruments) with a bandwidth of 20 Hz to 150 kHz and a 192 kHz sampling rate or a self-calibrating, four-channel Soundtrap with a 20 Hz to 90 kHz bandwidth and a 192 kHz sampling rate with a High Tech Inc., high frequency hydrophone (2 Hz to 125 kHz frequency response).

Recordings were analyzed in Raven Pro 1.5, (Window type Hann, 2100 samples; DFT: 4096 samples, Hop Size 1050 samples; 50 percent overlap; Frequency y-axis: 0–50.0 kHz; Time x-axis 0–5.0 seconds). A researcher identified and boxed all whistle productions throughout the recordings and subsequently saved each whistle as an individual.wav file (16 bit; 192 kHz sampling rate) to a ‘vocal catalog’ that is maintained for each dolphin at the MMP.

**Fig 1:**
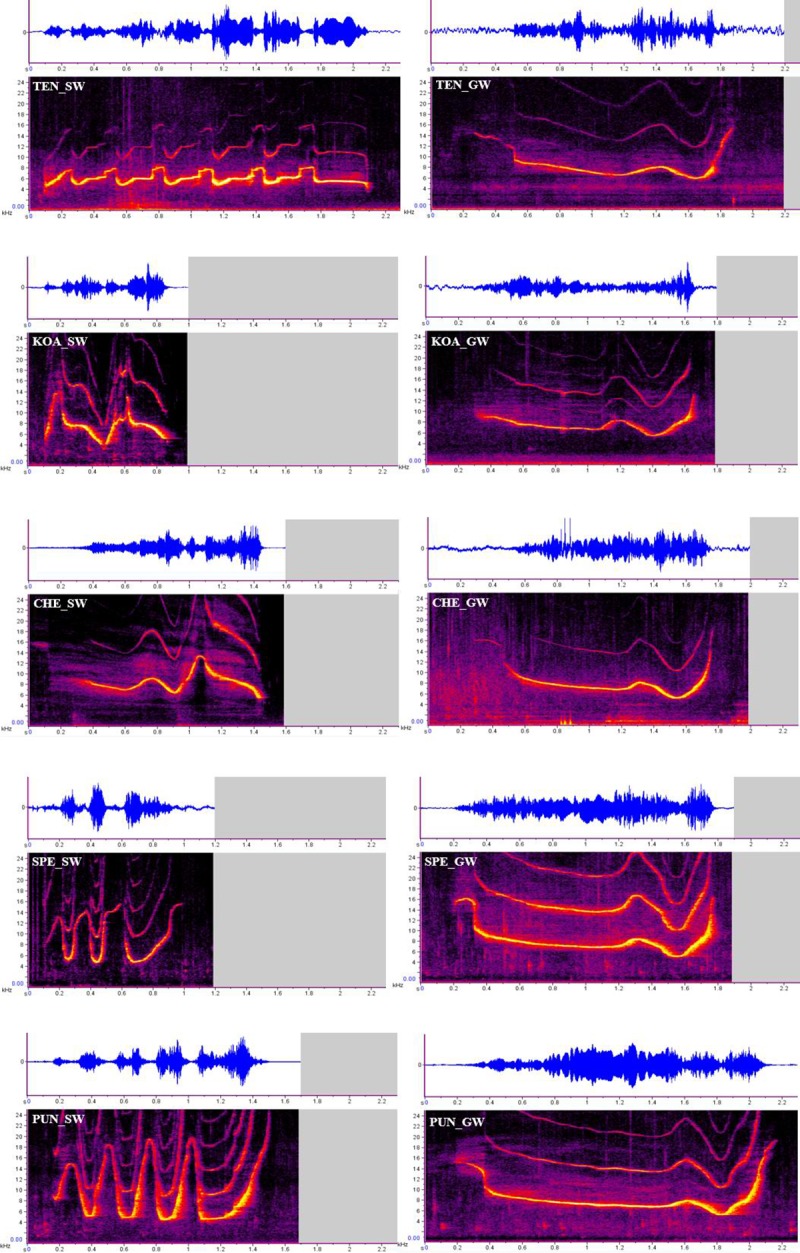
Signature and group whistle spectrograms. Time in seconds is on the x-axis with frequency in kilohertz (kHz) on the y-axis. The top panel depicts the waveform of the whistle over time with relative amplitude represented on the y-axis.

Janik et al., [40] defined whistles that have the same frequency modulation pattern over time (i.e., whistle contour) to be considered the same whistle type. We identified the whistle contour that was produced the majority of the time during periods of separation [35] and classified it as that dolphin’s signature whistle (SW). All other whistle types were considered non-signature whistles or in some cases copies of other dolphin’s signature whistles. It became apparent that there was a non-signature whistle type that was produced by a number of our focal animals and emitted more often than any other non-signature whistle recorded. This whistle type was termed the group whistle (GW). B. Jones assessed all whistles recorded from the focal animal with good signal to noise ratio and assigned the contour by visual inspection [44] as either a signature whistle exemplar, a group whistle exemplar, or a non-signature whistle (i.e., all non-signature and non-group whistle contours). Members of the focal group produced their individual signature whistles an average of 43% +/- 12%. The group whistle contour was produced an average of 22% +/- 9% of the time. All other non-signature whistle types combined made up an average of 35% +/- 9% of their repertoire. See Fig 1 for a spectrogram and waveform of each of the 10 whistle types included in this study (i.e., 5 signature whistles, and 5 group whistles).

When available, 25 signature whistles and 25 group whistles (minimum 17) were collected and included for each dolphin (N = 5). For dolphins that produced either their signature whistle contour and/or the group whistle contour more than 25 times with good signal to noise ratio, a random number generator chose which whistle exemplars were used in the present study. This prevented observer bias in which whistle productions were included. A second researcher verified the contour groups to ensure inter-observer agreement between contour type assignments. There were zero disagreements between the two observers as to whistle type categorization for the 241 whistles included in this study.

In order to assess whistle similarity we used both naïve observers, and discriminant function analyses. First, three randomly selected signature whistles and three randomly selected group whistles from each dolphin were selected. These were then presented as pairwise comparisons to 16 naïve observers. The raters had experience in some regard with dolphins and understood that they were looking at visual representations of dolphin whistles, but had no experience with these five dolphins’ whistle repertoires. Observers were asked to rate the two whistles (see spectrogram settings above) in terms of similarity of contour shape on a scale of 1 (not similar at all) to 5 (exactly the same). They were instructed to ignore other details such as amplitude of the whistle and background noise (depicted by darkness of color), and the presence or absence of harmonics (i.e., repetitions of the whistle contour at frequency intervals above each contour). Each observer rated one whistle from each dolphin to every other dolphin for; signature-to-signature whistles, signature-to-group whistles, and group-to-group whistles for a total of 55 comparisons. This provided us with seven categories of comparisons (see Table 2 for definitions). The comparisons of one dolphin’s signature whistle to a different dolphin’s group whistle, and a dolphin’s group whistle compared to another dolphin’s signature whistle were both omitted from subsequent analyses (see S1 Appendix for all pairwise comparison means) as they did not pertain to the present research questions.

**Table 2:** Definitions of each of the comparison types used throughout the subsequent analyses.

Comparison Type	Definition
**Dolphin’s SW to Own SW**	Dolphin A’s signature whistle contour compared to other iterations of Dolphin A’s signature whistle by Dolphin A
**Dolphin’s SW to Others’ SW**	Dolphin A’s signature whistle contour compared to each of the other dolphin’s (i.e., Dolphin B, C, D & E) signature whistles
**Dolphin’s GW to Own GW**	Dolphin A’s group whistle contour compared to other iterations of Dolphin A’s group whistle by Dolphin A
**Dolphin’s GW to Others’ GW**	Dolphin A’s group whistle contour compared to each of the other dolphin’s (i.e., Dolphin B, C, D & E) group whistles
**Dolphin’s SW to Own GW**	Dolphin A’s signature whistle contour compared to the group whistles also produced by Dolphin A
Dolphin’s SW to Others’ GW *Not Included	Dolphin A’s signature whistle contour compared to each of the other dolphin’s (i.e., Dolphin B, C, D & E) group whistles *Not Included
Dolphin’s GW to Others’ SW *Not Included	Dolphin A’s group whistle contour compared to each of the other dolphin’s (i.e., Dolphin B, C, D & E) signature whistles *Not Included

Throughout the results and discussion we refer to the comparison type abbreviation. *Not Included categories were comparisons that were made in order to provide all pairwise comparisons, but the comparison types were not part of our research questions and thus were not used in the following analyses. See S1 Appendix for mean values for all comparisons made.

To ensure observers understood the task, and were attentive throughout the process, 5 additional comparisons which showed two of the exact same spectrograms side by side were interspersed with the comparisons described above. To be included in analyses, observers had to rate at least three of the five comparisons as a “5 (exactly the same). Three of the 16 observers did not meet this requirement and as such were removed from analyses.

Krippendorf’s alpha was computed using the Kalpha Macro in SPSS 24 to calculate inter-rater reliability. Krippendorf’s alpha is a measure of reliability for multiple raters with 1.00 being perfect agreement and 0.00 being the absence of agreement [45]. The thirteen raters had an inter-rater reliability score of 0.72.

To examine whether the group whistle contours maintained identity information the specific acoustic parameters of each whistle were analyzed. The producer’s name for the whistle contours were dummy coded to avoid bias. Each whistle.wav file was then opened using Pamguard 1.15.15 CORE open source software [46] on a Microsoft Surface Book 2. Using the Rocca plugin [47], the.wav file was uploaded and the fundamental frequency contour of each whistle was traced using a fine tipped stylus pen. Rocca extracts the frequency by time contour and provides the user with 52 acoustic parameters of the contour for subsequent statistical analyses. This process was utilized for both the signature and group whistles for each dolphin.

All statistical analyses were run using IBM SPSS statistics 24. When Levene’s statistic found unequal variances between groups, Brown-Forsyth and Games-Howell statistics were used as robust tests for the ANOVA and post hoc tests respectively. For both the signature and the group whistle contours we chose six commonly used whistle characteristics (maximum frequency, minimum frequency, beginning frequency, end frequency, median frequency, and duration; Table 3) to analyze the main features of the whistle contours. These six parameters were used as the independent variables in a discriminant function analysis (DFA). The DFA was used to assess what factors allow us to best classify the whistles into groups (i.e., dolphin A SW, dolphin A GW, dolphin B SW, dolphin B GW, etc.) based on the six extracted parameters (Table 3). Frequency range and frequency center were not included in the DFA as they were too highly correlated to the included frequency parameters and failed the tolerance test (tolerance = 0.00). Removing these two parameters ensured that there were not redundancies and the multicollinearity assumption was not violated [48]. The factor loadings with eigenvalues > 1 (i.e., 3 factors) were used as a standardized representation of the location of each whistle contour in 3D space. The distance formula:

$$D=\sqrt{\left(x_{2}-x_{1})+(y_{2}-y_{1})+(z_{2}-z_{1}\right)}$$

was used to calculate the distance (D) of each whistle (x_1_, y_1_, z_1_) from the centroid of the whistle owner and also to the centroid of each other dolphin whistle type (x_2_, y_2_, z_2_). Mean distance was used as the similarity score parameter. Univariate ANOVAs were used to measure differences between similarities of whistle types by owner (e.g., distance between an owners SW and another dolphins SW compared to the distance between an owner’s SWs).

**Table 3:** Definitions of the six whistle characteristics included in the discriminant function analysis.

Whistle Characteristic	Definition
**Maximum Frequency**	Highest frequency of the whistle contour (kHz)
**Minimum Frequency**	Lowest frequency of the whistle contour (kHz)
**Beginning Frequency**	Frequency of the first point of the whistle contour (kHz)
**End Frequency**	Frequency of the last point of the whistle contour (kHz)
**Median Frequency**	The frequency at which half of the frequencies of the whistle contour lie above and half of the frequencies of the whistle contour lie below (kHz)
**Duration**	The time at the end point of the whistle contour minus the time at the beginning point of the whistle contour (s)

## Results

### Observer whistle contour similarity scores

Krippendorf’s alpha showed good inter-rater agreement for ten naïve human raters (α = 0.717; CI 0.703–0.731) [45,49,50]. See S1 Appendix for the mean and standard deviations of the similarity scores for all pairwise comparisons.

**Fig 2:**
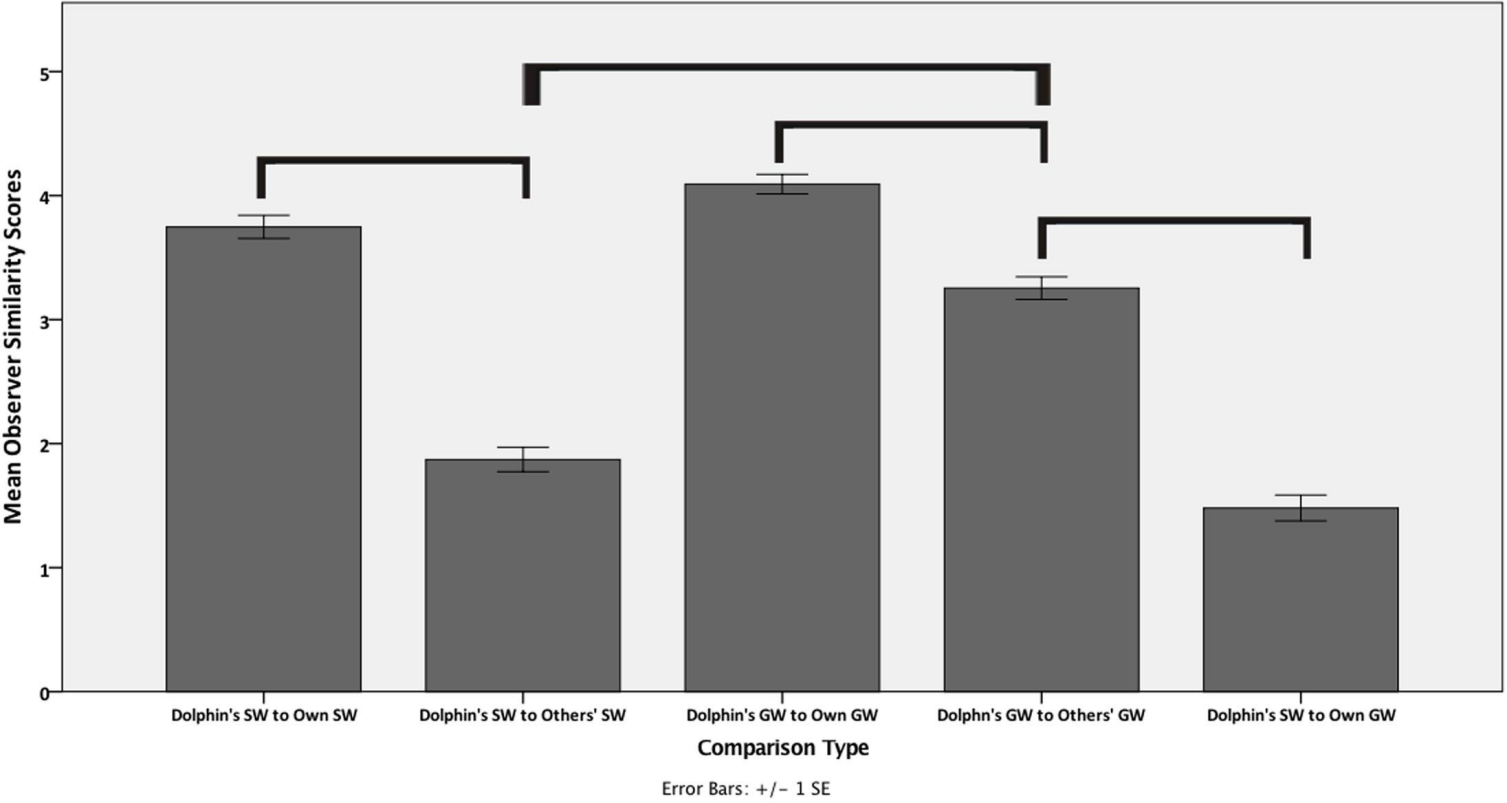
Mean similarity scores of overall contour shape. The x-axis denotes the comparison type and the y-axis shows the mean +/- 1 standard error of the similarity score generated by the naïve observers. The higher the similarity score, the more similar the whistle types were considered. The brackets indicate statistically significant differences between comparison types. We only include brackets for the comparisons of interest for our research questions above; therefore, two categories may be statistically different (e.g., Dolphins SW to Others’ SW and Dolphin’s GW to Own GW) but were not relevant to the current study.

A univariate ANOVA found an omnibus main effect of whistle comparison type on the mean similarity score (Brown-Forsythe: *F*_4, 454_ = 113.35, *P* = 0.000). Consistent with the signature whistle hypothesis, a dolphin’s SW to its own SW showed a high mean similarity score (mean (M) + standard error (SE) = 3.75 + 0.09, standard deviation (SD) = 0.89). A dolphin’s GW was also rated as highly similar to other renditions of that dolphin’s own GW (M + SE = 4.09 + 0.08; SD = 0.63). These results continued to support the signature whistle hypothesis in that the mean similarity score of a dolphin’s SW to its own SW (M + SE = 3.75 + 0.09; SD = 0.89) was significantly higher than a dolphin’s SW to other dolphins’ SWs (M + SE = 1.87 + 0.10; SD = 1.07) (*P* = 0.000). It further suggests that non-signature whistle types can also be highly stereotyped as the mean similarity score of a dolphin’s GW to its own GW (M + SE = 3.75 + 0.09, SD = 0.49) was significantly higher than a dolphin’s GW to other dolphins’ GWs (M + SE = 3.25 + 0.09, SD = 1.04) (*P* = 0.000) (Fig 2).

The shared whistle contour hypothesis was also supported as a dolphin’s GW compared to other dolphins’ GWs (M + SE = 3.25 + 0.09; SD = 1.04) was significantly more similar than both the dolphin’s SW to other dolphins’ SWs (M + SE = 1.87 + 0.10; SD = 1.07) and the dolphin’s GW compared to its own SW (M + SE = 1.48 + 0.11; SD = 0.75) (*P =* 0.000; *P* = 0.000). In fact, this was the highest rated similarity among comparisons besides the dolphin’s SW to its own SW and the dolphin’s GW to its own GW (Fig 2). The dolphin’s GW to its own GW (M + SE = 4.09 + 0.08; SD = 0.63) was still significantly higher (*P* = 0.000) than the dolphin’s GW to other dolphins’ GWs (M + SE = 3.25 + 0.09; SD = 1.04) suggesting that the group whistle contour still maintains some individual differences between dolphins.

### Whistle parameter analysis and classification

A discriminant function analysis found three discriminant functions with eigenvalues greater than 1. Function one explained 62% of the variance, canonical R^2^ = 0.92, the second explained 23% of the variance, canonical R^2^ = 0.81, and the third explained 12% of the variance, canonical R^2^ = 0.69 (total cumulative variance accounted for = 96%). Together the factors significantly predicted group membership; Wilks’ Lambda = 0.003, χ^2^ (54) = 1370.174, *P* = 0.000.

**Fig 3:**
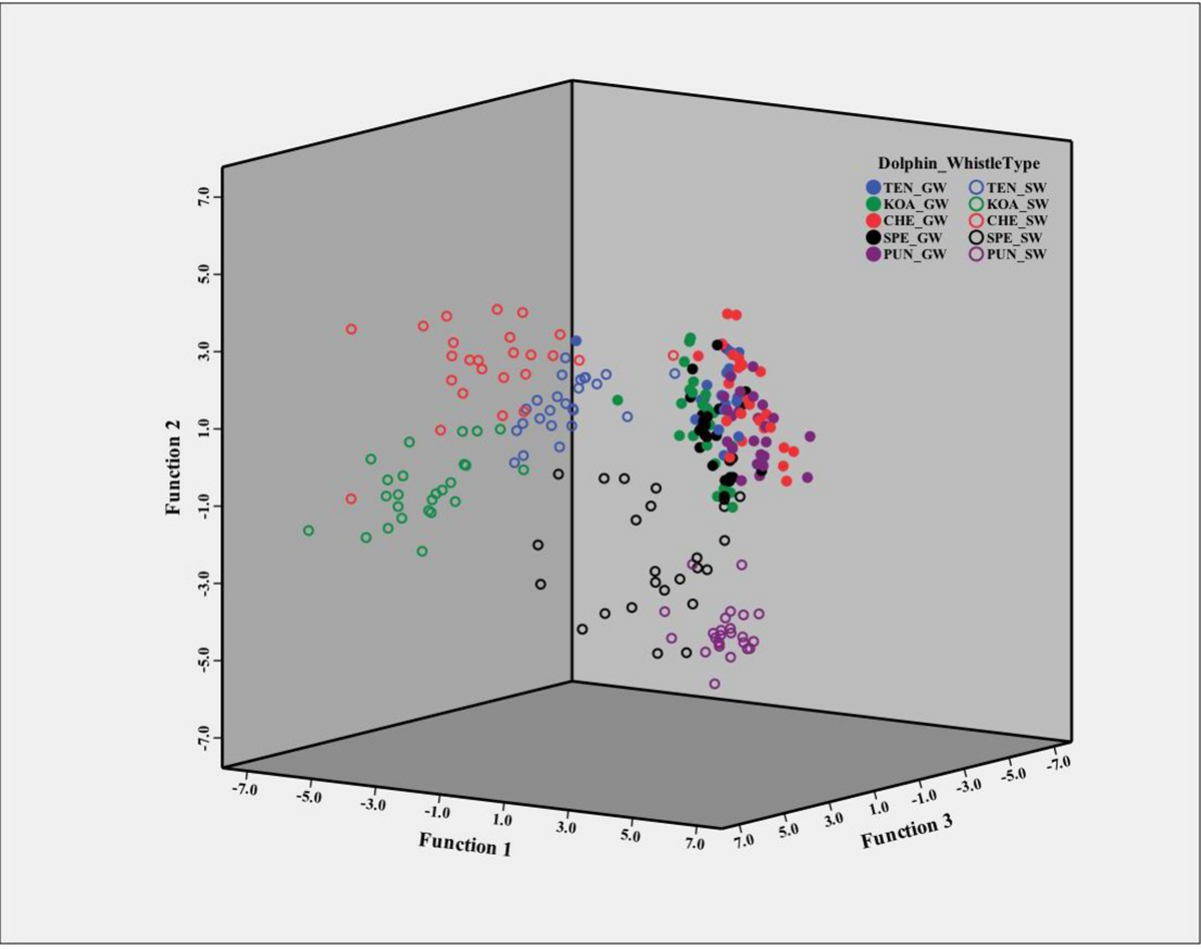
3D scatter plot comparing each dolphin’s GWs and SWs to each other’s. Each dolphin is represented by a color, and their signature whistle (SW) represented by an open circle while their group whistles (GW) denoted by a filled circle. The three factors with eigenvalues greater than 1 are represented on the x, y and z-axes. The whistles are plotted based on their scores (-7 –+7) on each of these factor s (x, y, z). The distance between two dots represents the similarity between their whistle parameters (see Table 1). Notice that the GWs of all five animals (filled circles) are clustered closely together suggesting that it is a shared whistle contour, whereas the SWs (open circles) cluster by dolphin.

The largest standardized factor loading for function one was end frequency at 0.819. Function two was categorized by multiple medium-sized loadings with minimum frequency being the largest at 0.495. Function three was most heavily characterized by the maximum frequency, loading at 0.603 (see Fig 3 for a plot of each whistle’s location based on the three significant functions). The function values for each group (i.e., dolphin_whistletype) for each of the three significant functions is reported in S2 Appendix. These group centroid values were coordinates used to calculate the distance of each individual whistle from the centroid for each dolphin whistle type.

The DFA correctly classified 72.6% (175/241) of the original whistles to the correct group (i.e., dolphin_whistletype), and when the leave-one-out classification procedure was used the classification accuracy was decreased to 69.3% (167/241). Signature whistles were correctly classified 86.4% of the time (108/125 whistles). Of the 17 whistles that were misclassified 12 were classified as another dolphin’s signature whistle. On only two occasions (0.8% of the cases) was a dolphin’s signature whistle misclassified as that dolphin’s group whistle contour and both of these times occurred with individual SPE.

The group whistles were easily differentiated from the signature whistles as 98.3% of the group whistles (114/116 whistles) were classified as such even if they were attributed to the wrong producer. The group whistles were designated to the wrong producer 49.1% of the time (57/116). The DFA was far more likely to successfully classify signature whistles to the correct owner’s (86.4%) than they were group whistles, but 50.9% of correct classifications of group whistles is still higher than expected by chance (i.e., 1/5 = 20%).

**Fig 4:**
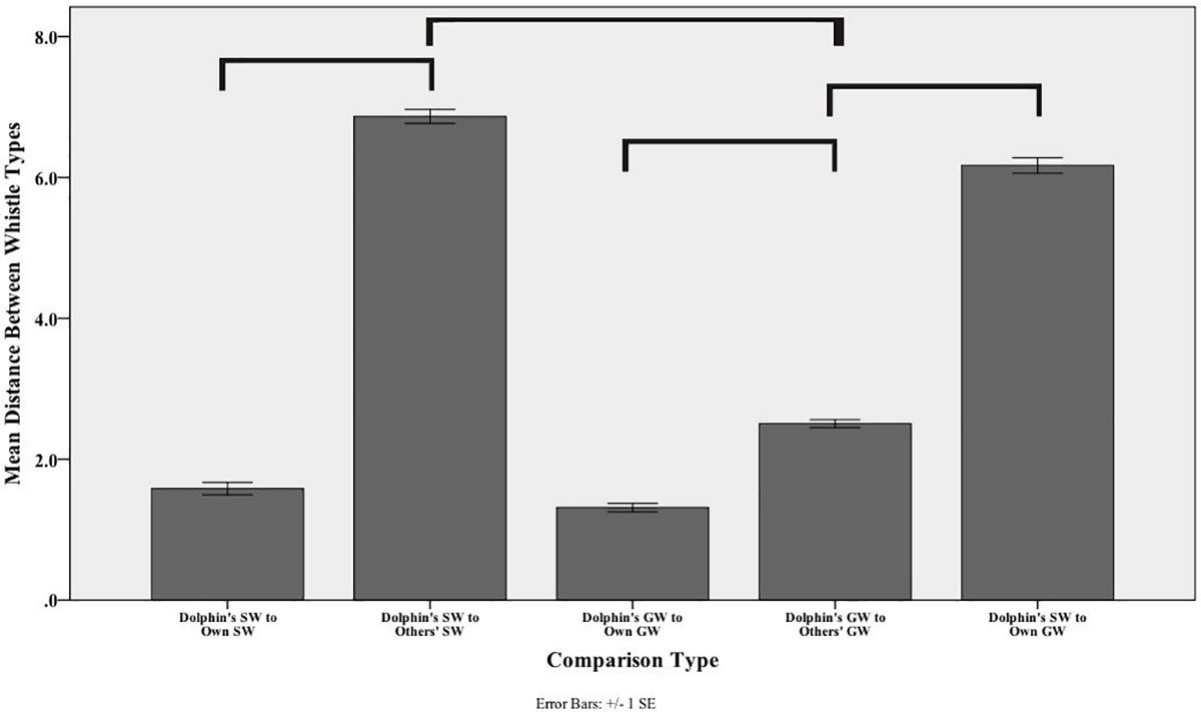
Distance between whistle to group centroid for each comparison type. The bars represent the mean distance (+/- 1 standard error) from one whistle type to the group centroid of another whistle type. These values were calculated from the DFA using five frequency parameters and the duration of the whistle contour. On the x-axis are the different comparisons that were assessed and on the y-axis is the mean distance in 3D space between two whistle types based on their loadings on the 3 significant factors (see S2 Appendix). The smaller the distance, the more similar the parameters of the two whistle types are. The brackets indicate statistically significant differences between comparison types. We only include brackets for the comparisons of interest for our research questions above, therefore two categories may be statistically different (e.g., Dolphin’s SW to Others’ SWs and Dolphin’s GW to Own GW) but were not important in the current study.

A univariate ANOVA found a significant difference between the mean distance of a whistle to the group centroids of each of the whistle types based on the comparison type; Brown-Forsythe *F*_4, 1442_ = 939.32, *P* = 0.000 (see Table 2 for comparison type descriptions). Fig 4 depicts the mean distance between a whistle to the group centroid of each of the different comparison types. The shorter the distance, the closer the whistle was to the group centroid when the three significant functions determined by the DFA were used as the x, y, and z-axes respectively.

Post hoc comparisons found that a dolphin’s SW to its own SW was significantly more similar (M + SE = 1.58 + 0.09; SD = 0.99) than a dolphin’s SW to other dolphins’ SWs (M + SE = 6.86 + 0.10; SD = 2.23), Games-Howell: *P* = 0.000. The group whistles showed the same trend as a dolphin’s GW compared to its own GW (M + SE = 1.32 + 0.06; SD = 0.67) was more similar than a dolphin’s GW compared to the other dolphins’ GWs (M + SE = 2.51 + 0.06; SD = 1.24), Games-Howell: *P* = 0.000.

As in the observer similarity score analyses, with the exception of the comparisons of a dolphin’s whistle type to other iterations of that same whistle from the same dolphin (i.e., dolphin’s SW to own SW and dolphin’s GW to own GW), the smallest mean distance between a whistle and another dolphin’s whistles was when comparing a dolphin’s GW to other dolphins’ GWs (M + SE = 2.51 + 0.06; SD = 1.24). This was significantly smaller than a dolphin’s own SW compared to other dolphins’ SWs (M + SE = 6.86 + 0.10; SD = 2.23), Games-Howell: *P* = 0.000.

A dolphin’s GW was also significantly more similar to the other dolphins’ GWs (M + SE = 2.51 + 0.06; SD = 1.24) than to its own SW (M + SE = 6.17 + 0.75; SD + 1.71), Games-Howell: *P* = 0.000. This suggests that two dolphin’s group whistles are more similar to one another than one dolphin’s own signature whistle is to their own group whistle.

## Discussion

Here we provide clear evidence of a mixed-sex group of bottlenose dolphins producing a stereotyped, shared whistle contour. Five dolphins produced the shared whistle contour on average 22.4% +/- 9.0% of the time during periods of isolation from group mates (i.e., free swimming in above ground pools). The group whistle was considered highly similar to other dolphins’ group whistles by both naïve observers and discriminant function analyses. Janik and Sayigh [51] suggest that the same whistle contour produced by different dolphins can still have individual variation in specific parameters even when the frequency modulation over time is relatively the same. The shared whistle contours described here follow this premise. Multiple iterations of the group whistle contour by the same dolphin were more similar than group whistles emitted by different dolphins. This was again found by two different methods and suggests that even shared whistle types have individually distinctive features.

These findings support the idea that it may be beneficial for animals to produce calls with both individual and group identifying information encoded [34]. Previous reports indicate that there are many potential benefits to having a group specific call (e.g., indicating group membership to both in-group and out-of-group members, increasing communication efficiency, maintaining group cohesion, managing social distance and signaling investment in the group relationships). Shared whistles may be useful for dolphins to coordinate behavior as King and Janik [43] recorded whistle matching during cooperative foraging events, and King et al [39] recorded non-signature whistle matching during cooperative mate guarding. Continued research on the behavioral and social context of shared whistle use will begin to shed light on why this group whistle may have formed, in what contexts it is emitted, and what, if any, benefits it may provide to users.

Signature whistles were highly stereotyped within an individual and were both rated and plotted as largely dissimilar to the other dolphins’ signature whistles. The cross-validated DFA was able to successfully classify the signature whistle to the correct owner 86.4% of the time (108/125 whistles) which was much greater than chance (i.e., 20%). The focal group produces their signature whistles an average of 43% of the time which is consistent with previous reports of signature whistle production in both captive and free-swimming bottlenose dolphins [36,52,53].

The DFA easily classified the group whistles from the signature whistles (98.3% of the time (114/116 whistles) even though about 49.1% of the time (57/116 whistles) it was assigned to the wrong producer. That said, the group whistle was correctly identified to the producer 50.9% of the time, (59/116 whistles) which is still greater than twice chance levels (20%). It is not surprising that there are subtle individual differences between the individual dolphin’s group whistle emissions. In studies of dolphins copying conspecifics’ signature whistles, researchers note that they do not typically produce exact matches [37]. The resulting hypothesis is that this may be beneficial so that the copy can be identified as such and therefore functions as a referential signal instead of being mistaken as a signature whistle production by the owner [37,42,51].

The focal dolphins included in this study have been living, working and traveling together in close proximity for a minimum of 21 years. Although this is an artificially formed group, the presence of a shared whistle type supports previous reports that animals that frequently associate may develop call types that are similar (i.e., whistle convergence/whistle accommodation) [12]. Smolker and Pepper [13] suggested that three allied bottlenose dolphins converged on a signature whistle contour. A more recent study of signature whistles in male allies by King et al., [54] did not find evidence of signature whistle convergence in the same population. Therefore, Smolker and Pepper [13] may have actually recorded a shared whistle contour from the three males, similar to the group whistle contour described here.

Unfortunately, longitudinal data is not available on the development of the shared whistle contour. Dolphins typically develop their signature whistles within the first year of life and the acoustic input from the environment and conspecifics can be critical to the vocal development of a calf [35,55–57]. Four of the six dolphins that have been recorded producing the shared whistle contour were acquired from the same location, but none of them were born during the same year. Although genetic relatedness between the animals in this group cannot be confidently ruled out the difference in origin sites, ages, and genetic lineages of the dolphins in the present group make it unlikely that this contour was part of each of their repertoires prior to residing in San Diego Bay. For example, KOA was born in Hawaii to a Mississippi-collected dam and an Indian River Lagoon-collected sire. He had no acoustic connection with any of the other animals in this group during his first year of life. Continued recordings of confirmed related and unrelated dolphins housed with the focal group will shed light on the origin and/or development of this shared whistle type and others.

The present data was recorded as part of a larger ONR funded project studying sound production and welfare in dolphins. This project allows opportunistic recordings of eight focal dolphins during isolation events. To date, we have recorded six of the eight members of this group producing the group whistle (CST was omitted because she has only been recorded producing it 3 times to date). It is interesting to note that the 5 dolphins that produce this whistle most often have been part of this team for the longest duration (minimum 21 years). We cannot say for certain how many other dolphins that currently reside with, or have previously been associated with the focal group members also produce this shared whistle. That said, we have recorded whistles during isolation events of 44 other members of the MMP population and have not yet recorded a group whistle contour from any dolphin outside this group. The current report serves as a minimum number of animals that share this whistle contour but may not represent the extent of the shared whistle contour’s use within this population.

In summary, a mixed-sex group of bottlenose dolphins that is housed in San Diego Bay, CA produce a shared whistle contour. This whistle contour is produced an average of 22% of the time, second only to the production of individual signature whistles (M = 43% of the time). Although the group whistle contours were highly similar, they still showed individual differences between dolphins. To date, very little is known about the prevalence and function of shared whistle types in bottlenose dolphins. This is an exciting new opportunity for bio-acousticians to study the pervasiveness and contextual function of shared whistle contours in dolphin groups. The presence of shared whistle repertoires may also shed light on association patterns and social relationships in dolphin groups that are less accessible to researchers.

## Supporting information

S1 Appendix Mean similarity scores. The mean similarity scores for all pairwise comparisons rated by ten naïve human observers on a 5-point scale (1 = not similar to 5 = the same). The contour names are given by a three letter publication name representing the dolphin that produced the sound _ whistle type (i.e., signature whistle (SW) or group whistle (GW).
(DOCX)

S2 Appendix Functions at group centroids. The group centroid of each of the whistle types (i.e., dolphin_whistletype) for the three significant functions used by the DFA. The group centroid values were used as coordinated (X, Y, Z) in order to calculate the distance of each individual whistle to each of the group centroids for additional analyses.
(DOCX)
