# Supplementary material for: Five members of a mixed-sex group of bottlenose dolphins share a stereotyped whistle contour in addition to maintaining their individually distinctive signature whistles
Source: PLoS One. 2020 May 22;15(5):e0233658. doi: 10.1371/journal.pone.0233658 (PMC7244121; doi:10.1371/journal.pone.0233658)
Supplement: S1 Appendix — The mean similarity scores for all pairwise comparisons rated by ten naïve human observers on a 5-point scale (1 = not similar to 5 = the same). The contour names are given by a three letter publication name representing the dolphin that produced the sound _ whistle type (i.e., signature whistle (SW) or group whistle (GW). (DOCX) [file pone.0233658.s001.docx]

**S1 Appendix. Mean similarity scores.** The mean similarity scores and standard deviations for all pairwise comparisons rated by thirteen naïve human observers on a 5-point scale (1 = not similar to 5 = the same). The contour names are given by a three letter publication name representing the dolphin that produced the sound _ whistle type (i.e., signature whistle (SW) or group whistle (GW)).

| Contour A | Contour B | Mean Similarity Score | Standard  Deviations |
| --- | --- | --- | --- |
| CHE_GW | CHE_GW | 3.7 | 0.67 |
| CHE_GW | CHE_SW | 1.8 | 1.03 |
| CHE_GW | KOA_GW | 1.9 | 0.99 |
| CHE_GW | KOA_SW | 1 | 0 |
| CHE_GW | PUN_GW | 2.6 | 1.07 |
| CHE_GW | PUN_SW | 1.3 | 0.95 |
| CHE_GW | SPE_GW | 4 | 0.82 |
| CHE_GW | SPE_SW | 1.1 | 0.32 |
| CHE_GW | TEN_GW | 2.6 | 0.84 |
| CHE_GW | TEN_SW | 1.1 | 0.32 |
| CHE_SW | CHE_SW | 4.4 | 0.70 |
| CHE_SW | KOA_GW | 1.1 | 0.32 |
| CHE_SW | KOA_SW | 2 | 1.15 |
| CHE_SW | PUN_GW | 2.4 | 1.07 |
| CHE_SW | PUN_SW | 2 | 0.94 |
| CHE_SW | SPE_GW | 2.5 | 1.12 |
| CHE_SW | SPE_SW | 1.2 | 0.63 |
| CHE_SW | TEN_GW | 1.1 | 0.32 |
| CHE_SW | TEN_SW | 1.4 | 0.70 |
| KOA_GW | KOA_GW | 4 | 0.67 |
| KOA_GW | KOA_SW | 1.2 | 0.42 |
| KOA_GW | PUN_GW | 4.1 | 0.57 |
| KOA_GW | PUN_SW | 1 | 0 |
| KOA_GW | SPE_GW | 2.4 | 0.70 |
| KOA_GW | SPE_SW | 1.1 | 0.32 |
| KOA_GW | TEN_GW | 3.7 | 0.95 |
| KOA_GW | TEN_SW | 1.3 | 0.67 |
| KOA_SW | KOA_SW | 3.2 | 1.03 |
| KOA_SW | PUN_GW | 1 | 0 |
| KOA_SW | PUN_SW | 1.1 | 0.32 |
| KOA_SW | SPE_GW | 1 | 0 |
| KOA_SW | SPE_SW | 1.4 | 0.52 |
| KOA_SW | TEN_GW | 1 | 0 |
| KOA_SW | TEN_SW | 2 | 0.94 |
| PUN_GW | PUN_GW | 4.6 | 0.52 |
| PUN_GW | PUN_SW | 1.1 | 0.32 |
| PUN_GW | SPE_GW | 3.6 | 1.07 |
| PUN_GW | SPE_SW | 1.1 | 0.32 |
| PUN_GW | TEN_GW | 3.3 | 0.82 |
| PUN_GW | TEN_SW | 1 | 0 |
| PUN_SW | PUN_SW | 4.2 | 0.63 |
| PUN_SW | SPE_GW | 1 | 0 |
| PUN_SW | SPE_SW | 3.3 | 1.25 |
| PUN_SW | TEN_GW | 1 | 0 |
| PUN_SW | TEN_SW | 1 | 0 |
| SPE_GW | SPE_GW | 3.9 | 0.74 |
| SPE_GW | TEN_GW | 3 | 1.05 |
| SPE_GW | TEN_SW | 1.2 | 0.42 |
| SPE_SW | SPE_SW | 3.9 | 0.57 |
| SPE_SW | TEN_GW | 1.1 | 0.32 |
| TEN_GW | TEN_GW | 4.3 | 0.48 |
| TEN_GW | TEN_SW | 1.2 | 0.42 |
| TEN_SW | TEN_SW | 3.5 | 1.08 |
