# Supplementary material for: Five members of a mixed-sex group of bottlenose dolphins share a stereotyped whistle contour in addition to maintaining their individually distinctive signature whistles
Source: PLoS One. 2020 May 22;15(5):e0233658. doi: 10.1371/journal.pone.0233658 (PMC7244121; doi:10.1371/journal.pone.0233658)
Supplement: S2 Appendix — The group centroid of each of the whistle types (i.e., dolphin_whistletype) for the three significant functions used by the DFA. The group centroid values were used as coordinated (X, Y, Z) in order to calculate the distance of each individual whistle to each of the group centroids for additional analyses. (DOCX) [file pone.0233658.s002.docx]

**S2 Appendix. Functions at group centroids.** The group centroid of each of the whistle types (i.e., dolphin_whistletype) for the three significant functions used by the DFA. The group centroid values were used as coordinated (X, Y, Z) in order to calculate the distance of each individual whistle to each of the group centroids for additional analyses.

| Functions at Group Centroids | | | | | |
| --- | --- | --- | --- | --- | --- |
| Dolphin_WhistleType |  | **Function** | | | |
|  |  | **1** |  | **2** | **3** |
| TEN_GW |  | 1.833 |  | 1.667 | -.360 |
| TEN_SW |  | -4.470 |  | .421 | -2.775 |
| KOA_GW |  | .559 |  | .635 | -1.641 |
| KOA_SW |  | -6.292 |  | -1.374 | .911 |
| CHE_GW |  | 3.424 |  | 1.806 | .361 |
| CHE_SW |  | -2.951 |  | 2.428 | 2.749 |
| SPE_GW |  | 1.580 |  | .539 | -.873 |
| SPE_SW |  | .651 |  | -2.465 | .644 |
| PUN_GW |  | 3.563 |  | 1.136 | .365 |
| PUN_SW |  | 2.713 |  | -4.235 | .438 |
